# Isolation of primary mouse retinal ganglion cells using immunopanning-magnetic separation

**Published:** 2012-12-03

**Authors:** Samin Hong, Yoko Iizuka, Chan Yun Kim, Gong Je Seong

**Affiliations:** Institute of Vision Research, Department of Ophthalmology, Yonsei University College of Medicine, Seoul, Korea

## Abstract

**Purpose:**

To establish an effective system for isolating primary retinal ganglion cells (RGCs) from newborn mice.

**Methods:**

The retinas were separated from enucleated eyeballs of Crl:CD-1 mice on postnatal day 1 to 4. RGCs were purified using three different methods, including two-step immunopanning (TSI), direct magnetic separation (DMS), and immunopanning-magnetic separation (IMS). Harvested cells were maintained for 24 h in a defined medium and then examined with immunocytochemistry, western immunoblotting, and real-time reverse transcription polymerase chain reaction (RT-PCR) for glial cell–specific glial fibrillary acidic protein (GFAP) and amacrine cell-specific syntaxin 1.

**Results:**

As determined with immunofluorescence staining, RGCs purified by TSI were sparsely mixed with GFAP-positive astrocytes, and RGCs isolated by DMS were frequently mixed with syntaxin 1-positive amacrine cells. However, RGCs collected by IMS were seldom contaminated by GFAP-positive or syntaxin 1-positive cells. On western immunoblots, TSI cells showed significant GFAP expression, and DMS cells showed apparent syntaxin 1 expression, but IMS cells did not. Results of the real-time RT–PCR showed a similar tendency to those of the immunocytochemistry and western immunoblots.

**Conclusion:**

Primary mouse RGCs were highly purified by the IMS method, combining the benefits of the TSI and DMS methods. This isolation method may provide a good experimental system for studying glaucoma in vitro.

## Introduction

Glaucoma is the second leading cause of vision loss worldwide [1]. As the loss of retinal ganglion cells (RGCs) is the main pathological process of glaucoma [2], many researchers have tried to better understand the mechanisms of RGC death. Although retinal explant culture and mixed retinal cell culture can represent the intraretinal microenvironment and reflect intercellular interactions between RGCs and other retinal cells [3-5], isolate RGC culture is more helpful for investigating primary RGC responses in certain circumstances.

Since Barres et al. [6] introduced the two-step immunopanning (TSI) method, it has been widely used to purify primary RGCs in vitro [7-10]. Using the first panning step, cells that react to antimacrophage antibody, which are presumed to be macrophages/microglia and endothelial cells, can be depleted from retinal cell suspension. In the second panning step, cells that have affinities to antithymocyte differentiation antigen 1 (Thy 1) antibody, which are presumed to be RGCs, can be selected from the remaining mixed cells. Though the purity of RGCs isolated by the TSI method has been reported to reach 99.5% [6], it is very complicated, and its yield varies.

To improve upon TSI, a magnetic cell sorter was applied to RGC purification [11]. Using anti-Thy 1 antibody and conjugated magnetic microbeads, RGCs are extracted from a mixed retinal cell suspension [12-15]. Although this direct magnetic separation (DMS) method is simpler and has a more stable yield than TSI, the purity of RGCs isolated by the DMS method is much lower than that of RGCs isolated by the TSI method [11,15].

In this investigation, to establish an effective system for isolating primary RGCs, intended specifically for use in samples from newborn mice, we evaluated the characteristics of RGCs purified by the TSI, DMS, and combined immunopanning-magnetic separation (IMS) methods.

## Methods

### Animals

A total of 27 pregnant Crl:CD-1 mice were purchased from Orientbio (Seongnam, Republic of Korea). Nine animals were used for immunocytochemistry, nine animals were used for western immunoblots, and nine animals were used for real-time reverse transcription-polymerase chain reaction (RT–PCR) experiments. In terms of mice pups, 387 newborn mice were euthanized by decapitation. All animals were treated in accordance with the ARVO Statement for the Use of Animals in Ophthalmic and Vision Research and the guidelines of the Institutional Animal Care and Use Committee. Great effort was made to minimize the number of animals euthanized and their suffering. Each following experiment was conducted in triplicate and repeated three times from different cell harvests.

### Retinal cell suspension

Retinal tissues were separated from the enucleated eyeballs of newborn mice on postnatal day 1 to 4 and incubated in calcium-free and magnesium-free Hank’s balanced salt solution (Life Technologies, Grand Island, NY) containing 5 mg/ml of papain, 0.24 mg/ml of L-cysteine, 0.5 mmol/l of EDTA, and 10 U/ml of DNase І for 20 min. The retinal cells were mechanically dissociated by gentle pipetting and collected as a suspension. About 1.5 million cells were collected per retina. Procedures were conducted at room temperature in a laminar flow hood.

### Two-step immunopanning

**Figure 1:**
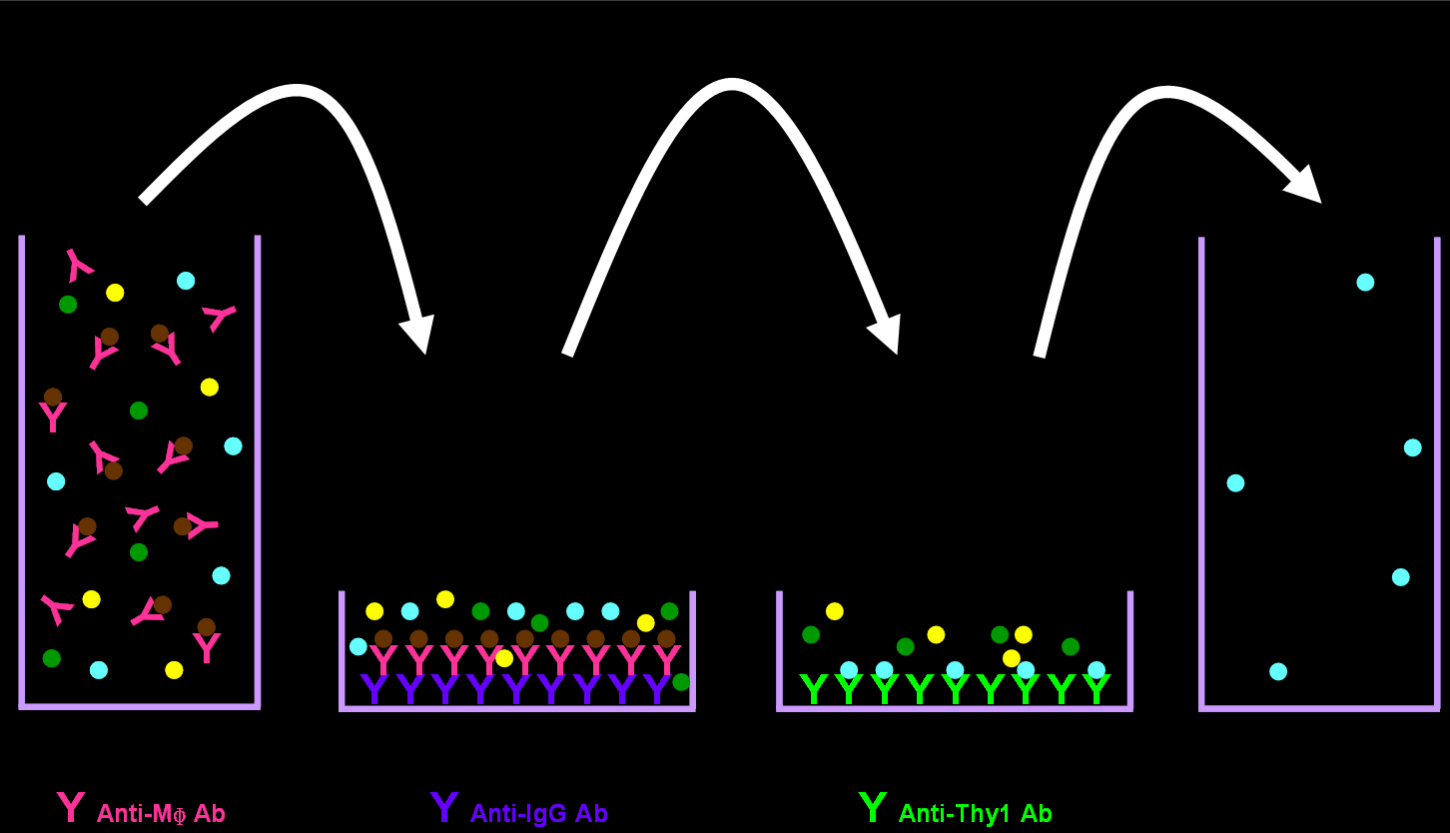
Two-step immunopanning method for isolating retinal ganglion cells. Retinal cell suspension is incubated with antimacrophage antibody (Anti-MΦ Ab) and treated in a Petri dish coated with antiimmunoglobulin G antibody (Anti-IgG Ab). Non-adherent cells are then treated in the second Petri dish coated with antithymocyte differentiation antigen 1 (Anti-Thy 1 Ab). Finally, the adherent cells are collected as retinal ganglion cells.

RGCs were isolated using the TSI method as previously described (Figure 1) [6]. Retinal cell suspension was incubated with rabbit antimouse macrophage antibody (1:50 dilution; Fitzgerald Industries International, Concord, MA) for 5 min. The suspension was treated in a 100-mm Petri dish coated with goat antirabbit antiimmunoglobulin G antibody (1:200 dilution; Southern Biotechnology Associates, Birmingham, AL) for 30 min. Non-adherent cells were then treated in a second 100-mm Petri dish coated with rat antimouse Thy 1.2 antibody (1:50 dilution; Abcam, Cambridge, MA) for 1 h. The adherent cells were collected as RGCs. All procedures were performed at room temperature.

### Direct magnetic separation

**Figure 2:**
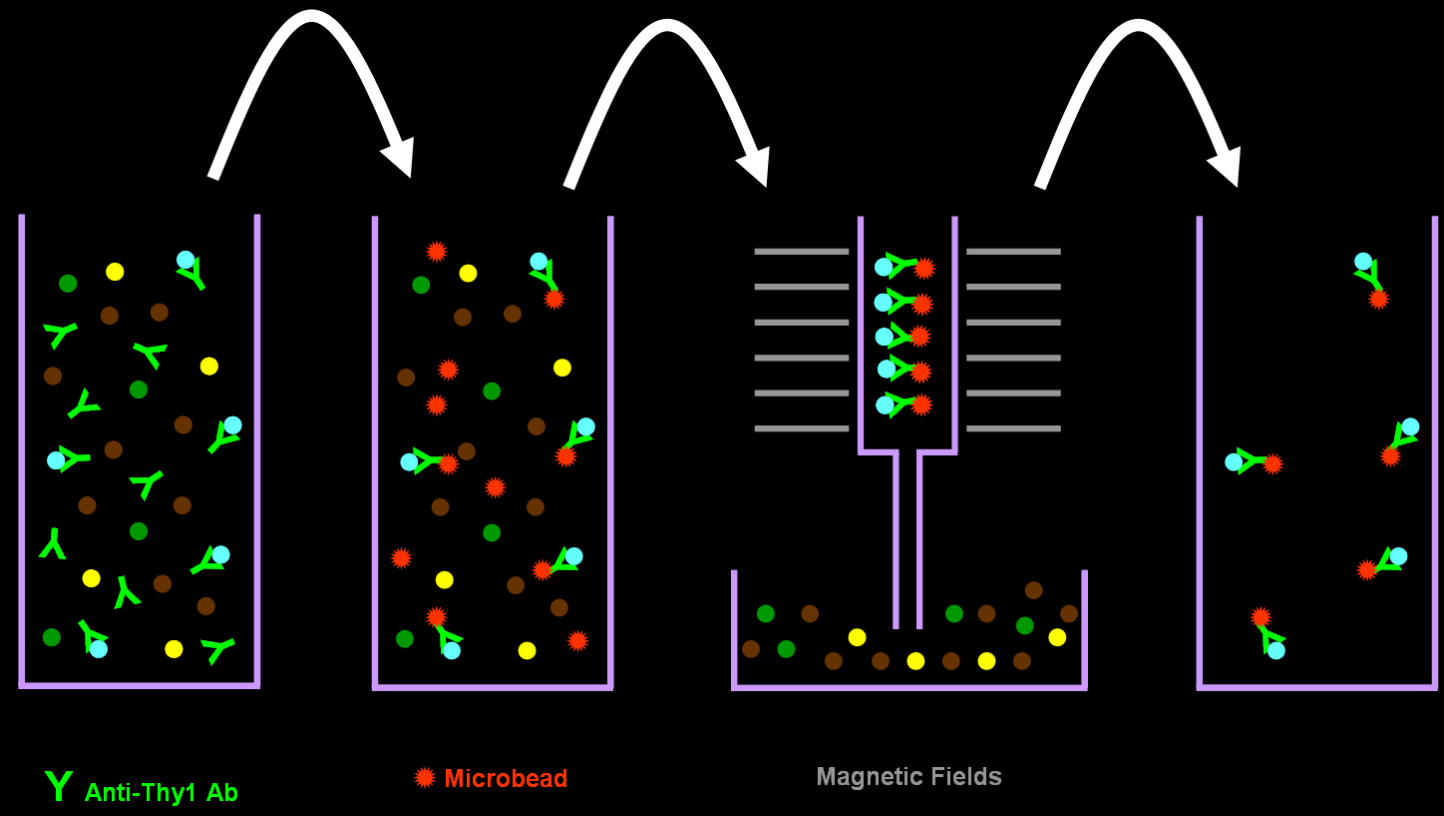
Direct magnetic separation method for isolating retinal ganglion cells. Retinal cell suspension is incubated with biotinylated anti-Thy 1 antibody and incubated with antibiotin magnetic MicroBeads. The cells are then applied onto a column placed in the magnetic field. Finally, the retained cells are eluted as a magnetic-labeled retinal ganglion cell fraction.

RGCs were purified using the DMS method as previously described (Figure 2) [11]. Retinal cell suspension was incubated with biotinylated rat antimouse Thy 1.2 antibody (1:20 dilution, Abcam) for 1 h at 37 °C. The cells were then incubated with MACS antibiotin MicroBeads (1:10 dilution; Miltenyi Biotec, Bergisch Gladbach, Germany) for 30 min at 4 °C. After careful washing, the cells were applied onto a MACS MS Column (Miltenyi Biotec) placed in a MiniMACS Separator (Miltenyi Biotec). The column was removed from the separator, and the retained cells were eluted as a magnetic-labeled RGC fraction.

### Immunopanning-magnetic separation

**Figure 3:**
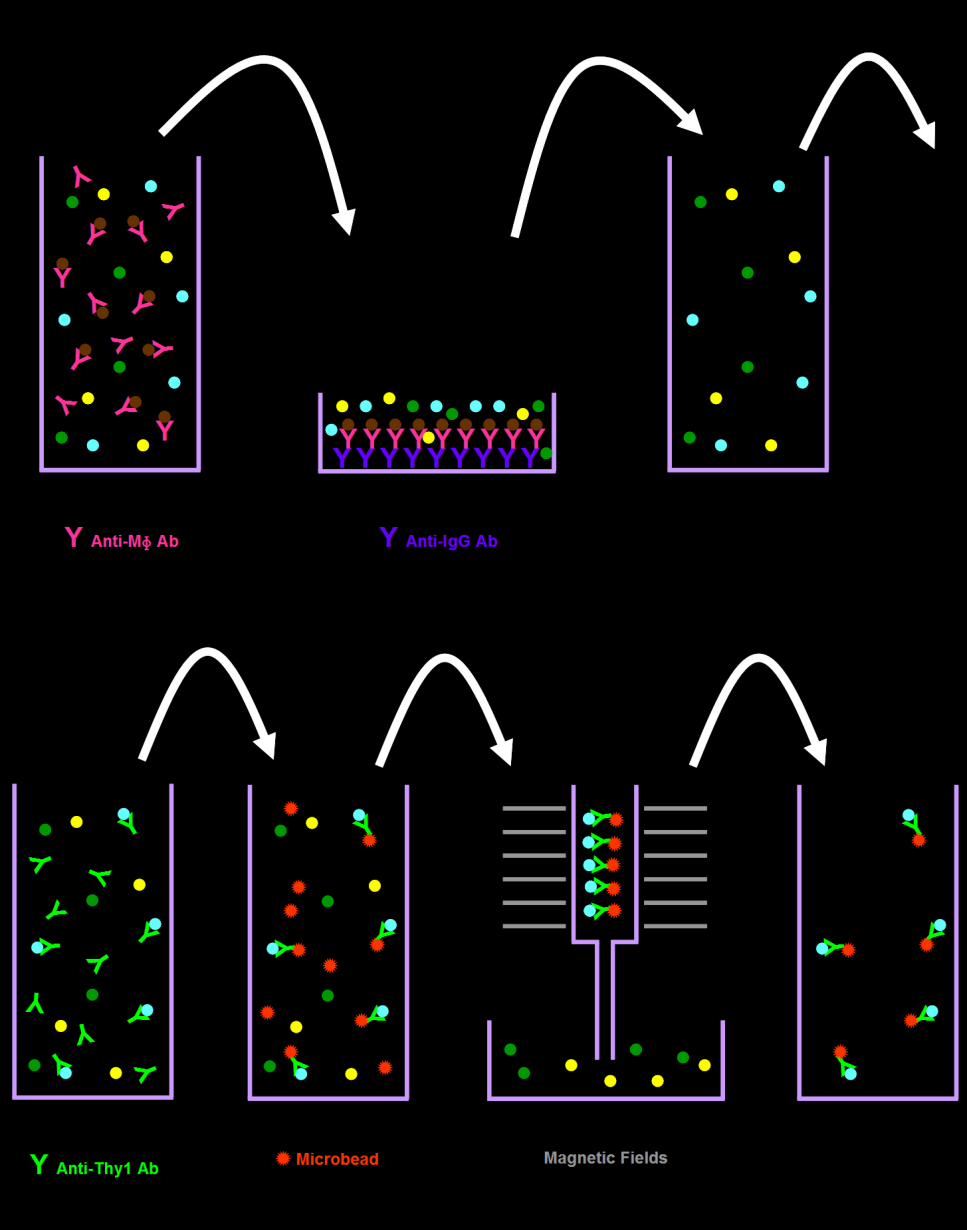
Immunopanning-magnetic separation method for isolating retinal ganglion cells. Retinal cell suspension is incubated with antimacrophage antibody and distributed over an antiimmunoglobulin G antibody-coated Petri dish. Non-adherent cells are treated with biotinylated anti-Thy 1 antibody and subsequently interacted with antibiotin magnetic MicroBeads. Finally, the magnetic-labeled retinal ganglion cells were collected using a column placed in the magnetic field.

RGCs were harvested using the IMS method (Figure 3). Briefly, similar to the first panning step of the TSI method, the retinal cell suspension was incubated with antimacrophage antibody and then distributed over an antiimmunoglobulin G antibody-coated Petri dish. Similar to the DMS method, non-adherent cells were treated with biotinylated anti-Thy 1.2 antibody and subsequently interacted with antibiotin MicroBeads. Finally, the magnetic-labeled RGCs were collected using a magnetic separating unit.

### Cell culture

The cells were grown in modified Politi’s medium [16,17]. The medium contained Dulbecco's modified Eagle's medium: nutrient mixture F-12 (Life Technologies) plus 10% fetal bovine serum (Life Technologies), insulin (20.0 mg/l), transferrin (20.0 mg/l), putrescine (64.4 mg/l), progesterone (25.2 μg/l), selenite (20.8 μg/l), hydrocortisone (72.4 μg/l), cytidine-5′-diphosphocholine (2.0 mg/l), cytidine-5′-diphosphoethanolamine (6.41 mg/l), brain-derived neurotrophic factor (80 μg/l), ciliary neurotrophic factor (80 μg/l), forskolin (4.0 mg/l), 100 U/ml penicillin, and 100 μg/ml streptomycin. Cells were seeded on the surfaces of 12-mm glass coverslips precoated with poly-L-ornithine and laminin. The seeding density was about 2.5×10^5^ cells per well. The cultures were incubated at 37 °C in humidified 5% CO_2_ and 95% air.

### Immunocytochemical staining

Twenty-four hours after seeding, cells were fixed with 4% paraformaldehyde for 30 min, treated with 0.1% Triton X-100 (Sigma-Aldrich, St. Louis, MO) in 0.1% Na-Citrate (Sigma-Aldrich) for 10 min, and then blocked with 2% bovine serum albumin (Sigma-Aldrich) for 1 h. Cells were incubated with astrocyte-specific antimouse glial fibrillary acidic protein (GFAP) antibody (1:100 dilution; Abcam) and amacrine-specific antimouse syntaxin 1 antibody (1:50 dilution; Santa Cruz Biotechnology, Santa Cruz, CA) overnight at 4 °C. They were then exposed to the corresponding fluorescent secondary antibodies (1:100 dilution) for 1 h at room temperature. Nuclei were counterstained with 4',6-diamidino-2-phenylindole (DAPI; Life Technologies). After a mounting medium was applied (Vectashield, Vector Laboratories, Burlingame, CA), four random fields were imaged under a fluorescence microscope to determine the RGC purity of each isolation method. The cells with fluorescence were manually counted at 400x magnification.

### Western immunoblots

To extract the total cell protein, cells were lysed in cell lysis buffer (50 mM Tris-HCl pH 7.4, 1% NP-40, 0.25% Na-deoxycholate, 150 mM NaCl, 1 mM EDTA, 10 mM Na_3_VO_4_, 50 mM NaF, 1 mM phenylmethanesulfonylfluoride, 1 μg/ml aprotinin, 1 μg/ml leupeptin, 1 μg/ml pepstatin) on ice for 30 min. Lysates were sonicated, and cell homogenates were centrifuged at 15,000 ×g for 10 min at 4 °C.

Protein concentrations in the resultant supernatants were determined with BCA protein assay (Thermo Fisher Scientific, Rockford, IL), and equal amounts of protein (10 μg) from each sample were boiled in Laemmli sample buffer and resolved by sodium dodecyl sulfate–polyacrylamide gel electrophoresis on 10% or 15% gels. The proteins were transferred to polyvinylidene fluoride membranes and incubated overnight with antibodies against either GFAP (Abcam), syntaxin 1 (Santa Cruz Biotechnology), or β-actin (1:1000 dilution; Santa Cruz Biotechnology). Immunoreactive bands were detected with horseradish peroxidase-conjugated secondary antibodies and were visualized with enhanced chemiluminescence.

### Real-time reverse transcription polymerase chain reaction

Total RNA was extracted using an RNeasy Mini Kit (Qiagen, Venlo, the Netherlands), and cDNAs were synthesized using the SuperScript III First-Strand Synthesis System (Life Technologies). Real-time PCR was performed with 20 ng of cDNA per reaction using 25 μl of iQ SYBR Green Supermix (Bio-Rad Laboratories, Hercules, CA) containing 500 nM of specific primers (Table 1) in the iCycler iQTM Real-Time PCR Detection System (Bio-Rad Laboratories). Using the SYBR Green data, a relative RNA ratio was calculated by dividing the value of each RGC purification method by the value of the entire retinal cell suspension.

**Table 1:** Primer sequences for real-time reverse transcription polymerase chain reaction.

Gene name	Type	Sequence
GFAP	Forward	5′-ACCGCATCACCATTCCTGTAC-3′
	Reverse	5′-TGGCCTTCTGACACGGATTT-3′
Syntaxin 1	Forward	5′-ACCGCTTCATGGATGAGTTC-3′
	Reverse	5′-GAGCTCCTCCAGTTCCTCCT-3′

GFAP=glial fibrillary acidic protein.

### Statistical analysis

RGC purity and quantitative RT–PCR data were expressed as the mean ± standard error of the mean (SEM) and compared with the Kruskal–Wallis one-way analysis of variance (ANOVA) using the PASW Statistics 18 for Windows, version 18.0.0 (SPSS, Chicago, IL). A p value of less than 0.05 was considered statistically significant.

## Results

### Retinal ganglion cells isolated with two-step immunopanning

**Figure 4:**
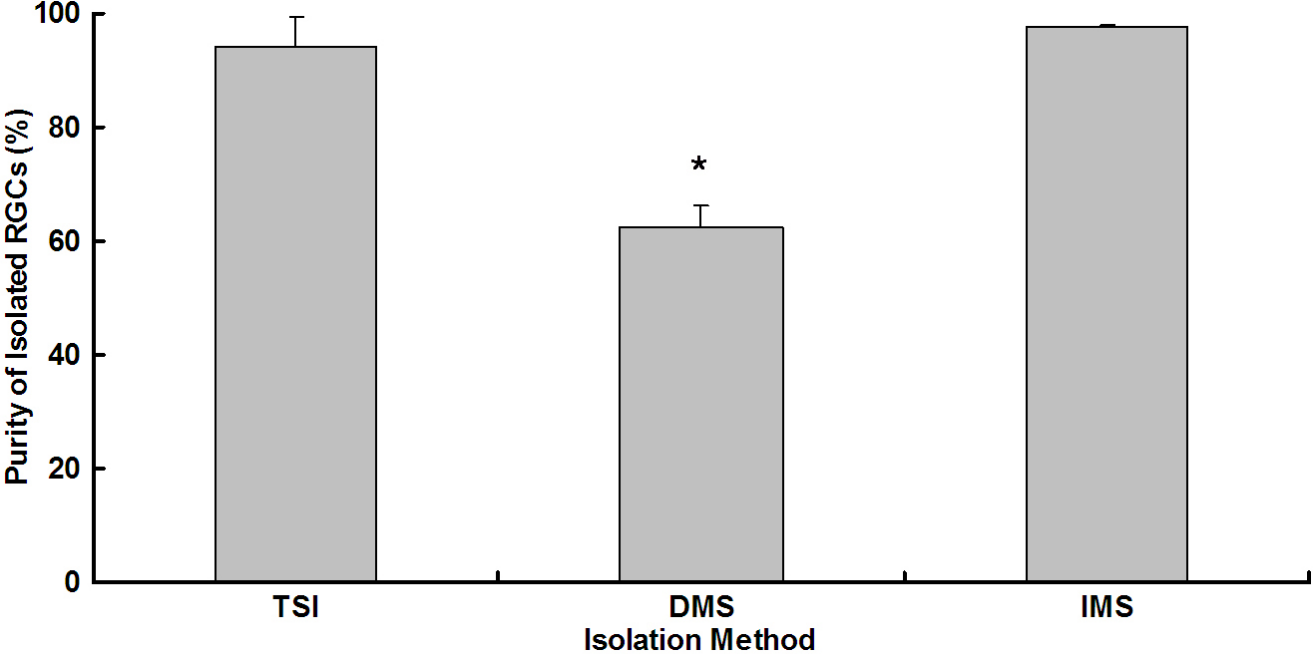
Purity of isolated retinal ganglion cells for three different methods, including two-step immunopanning, direct magnetic separation, and immunopanning-magnetic separation. Data were expressed as the mean±SEM (n=9 for each method).*p=0.023.

**Figure 5:**
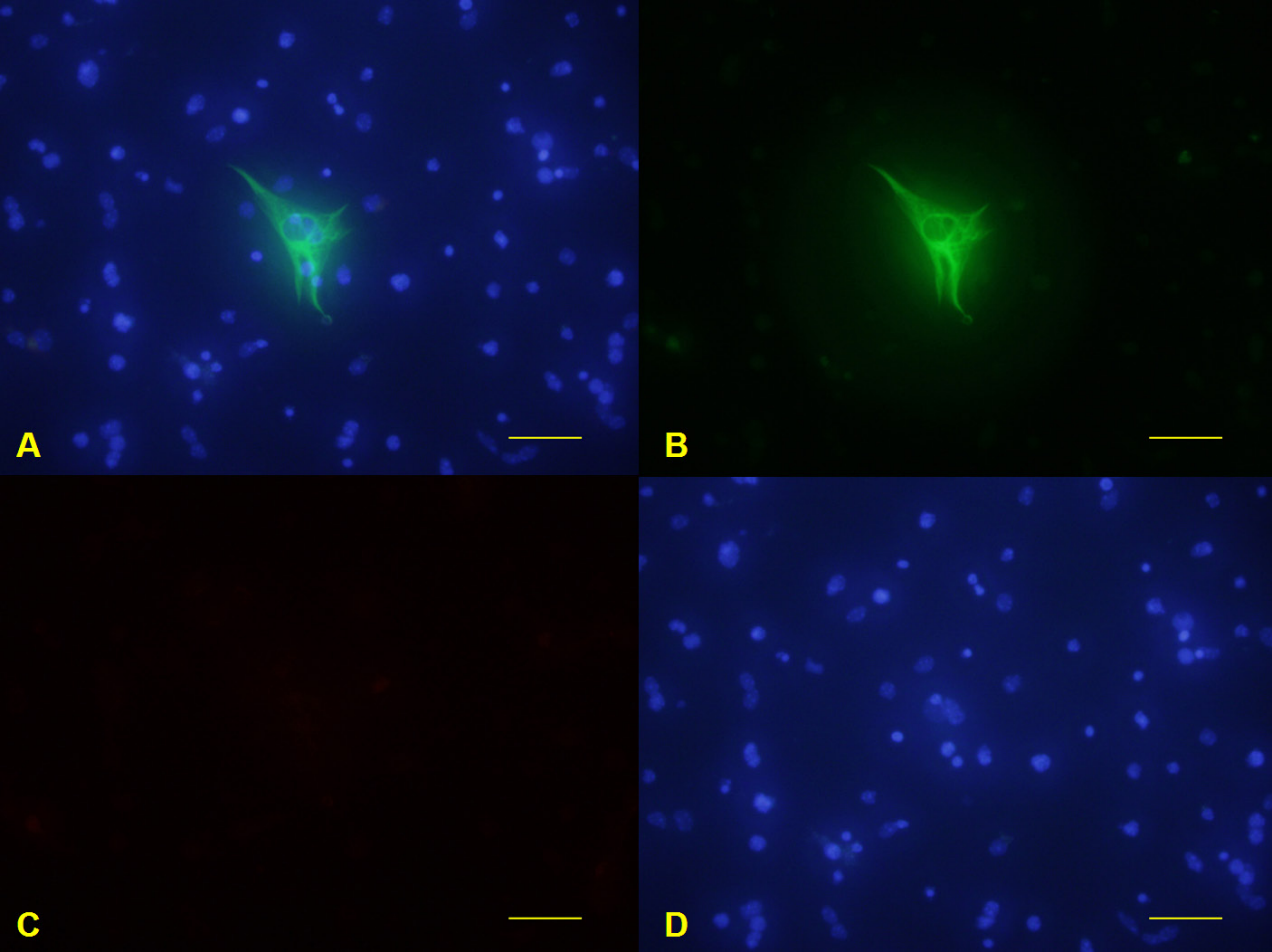
Primary mouse retinal ganglion cells isolated with two-step immunopanning. Glial fibrillary acidic protein–labeled cells were detected with green fluorescence (**B**). Syntaxin 1-labeled cells were detected with red fluorescence (**C**). 4',6-diamidino-2-phenylindole nuclear staining was detected with blue fluorescence (**D**). Merge image was constructed (**A**). Scale bars: 100 μm.

The purity of RGCs isolated by the TSI method was 94.06±5.13%, as determined by immunofluorescence staining (Figure 4 and Figure 5). Most of the harvested cells had small to medium round cell bodies, and some extended fine neurites. These GFAP-negative, syntaxin 1-negative cells were regarded as RGCs. Among RGCs, large star-shaped cells were sparsely found (Figure 5), and these GFAP-labeled cells appeared to be astrocytes.

### Retinal ganglion cells isolated by direct magnetic separation

**Figure 6:**
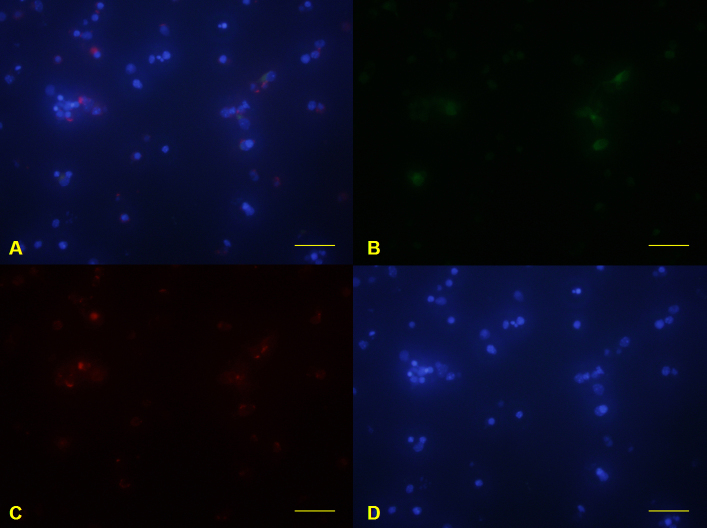
Primary mouse retinal ganglion cells isolated by direct magnetic separation. Glial fibrillary acidic protein–labeled cells were detected with green fluorescence (**B**). Syntaxin 1-labeled cells were detected with red fluorescence (**C**). 4',6-diamidino-2-phenylindole nuclear staining was detected with blue fluorescence (**D**). Merge image was constructed (**A**). Scale bars: 100 μm.

The purity of RGCs isolated by the DMS method was 62.45±3.84%, as determined by immunofluorescence staining (Figure 4 and Figure 6). The RGC purity for DMS was significantly lower than the other two methods (p=0.023). Although the greater part of the collected cells was small to medium round cells, several small cells showed immunoreactivity to syntaxin 1 (Figure 6). These syntaxin 1-positive small cells were identified as amacrine cells.

### Retinal ganglion cells isolated by immunopanning-magnetic separation

**Figure 7:**
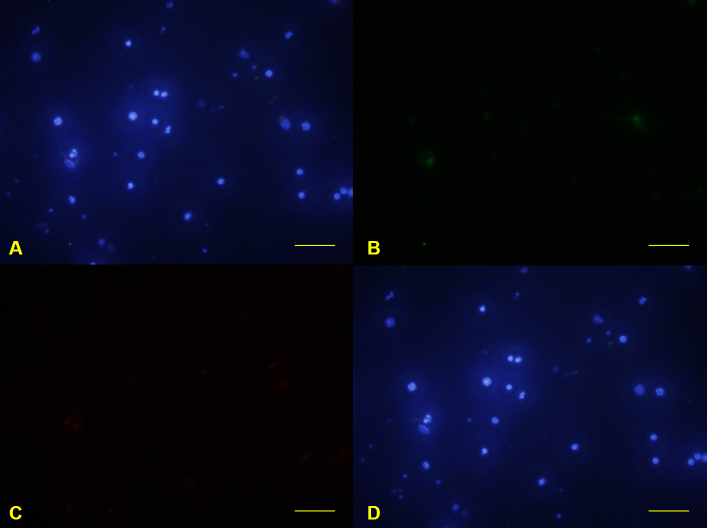
Primary mouse retinal ganglion cells isolated by immunopanning-magnetic separation. Glial fibrillary acidic protein–labeled cells were detected with green fluorescence (**B**). Syntaxin 1-labeled cells were detected with red fluorescence (**C**). 4',6-diamidino-2-phenylindole nuclear staining was detected with blue fluorescence (**D**). Merge image was constructed (**A**). Scale bars: 100 μm.

The purity of RGCs isolated by the IMS method was 97.72±0.28%, as determined with immunofluorescence staining (Figure 4 and Figure 7). Nearly all harvested cells were regarded as RGCs. Large star-shaped GFAP-positive astrocytes were not found in the entire field, and small round syntaxin 1-positive amacrine cells were seldom observed (Figure 7).

### Western immunoblots

**Figure 8:**
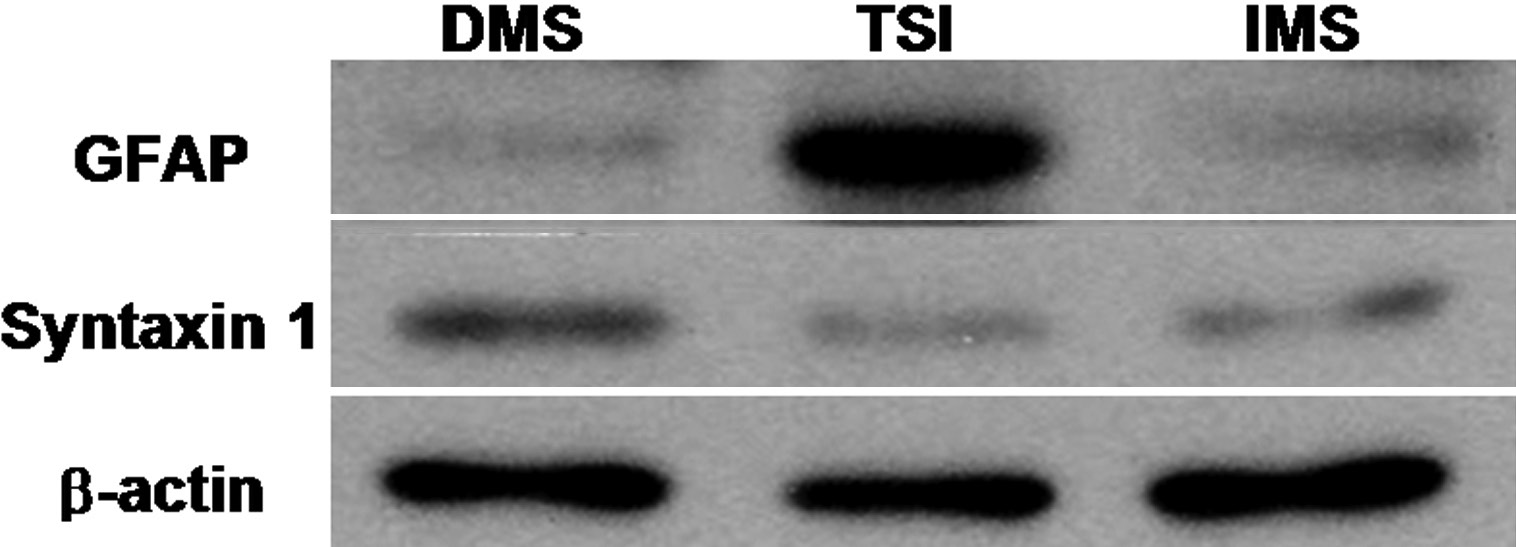
Representative western immunoblots for glial fibrillary acidic protein, syntaxin 1, and β-actin. Primary mouse retinal ganglion cells were isolated using three different systems, including two-step immunopanning, direct magnetic separation, and immunopanning-magnetic separation.

To verify the characteristics of contaminated cells during RGC purification, cell type–specific markers were evaluated using western immunoblots (Figure 8). Protein extracted from RGCs purified by TSI contained a lot of GFAP protein, but the protein extracted from RGCs purified by DMS and IMS did not. Regarding syntaxin 1, DMS-isolated RGCs contained a significantly greater amount of syntaxin 1 protein compared to TSI/IMS-isolated RGCs.

### Real-time reverse transcription polymerase chain reaction

**Figure 9:**
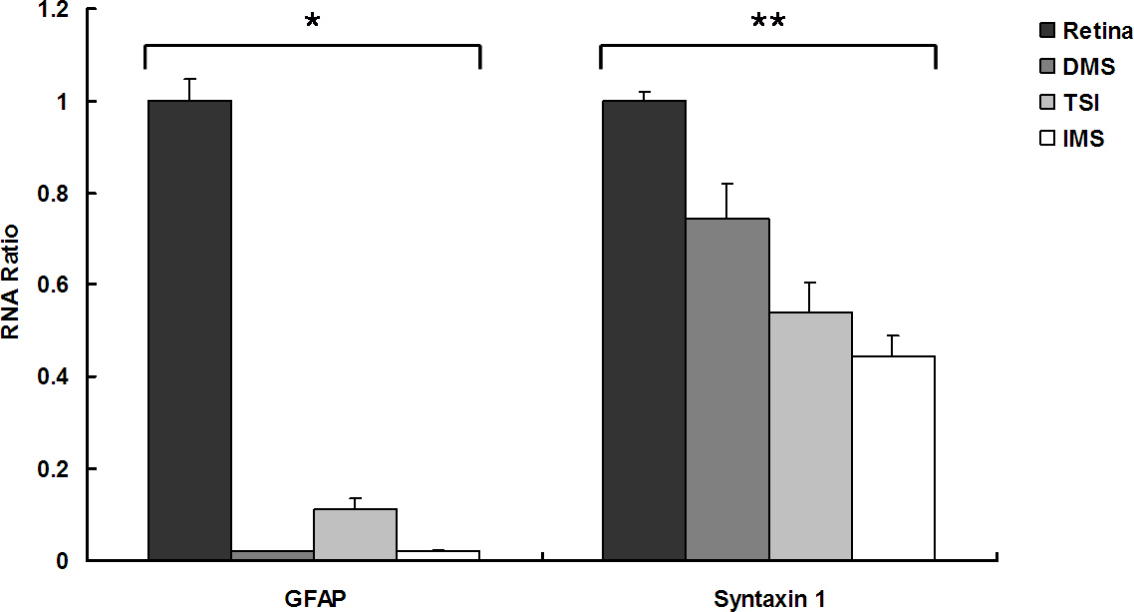
Quantitative data of real-time reverse transcription polymerase chain reaction (RT-PCR) for glial fibrillary acidic protein (GFAP) and syntaxin 1. A relative RNA ratio was calculated by dividing the value of each purification method by the value of whole retinal cell suspension. Data were expressed as the mean±SEM. (n=9 for each method). The p value for GFAP and syntaxin 1 was 0.021* and 0.022**, respectively.

The quantitative data of real-time RT–PCR for GFAP and syntaxin 1 were calculated with a relative RNA ratio, which was calculated by dividing the value of each RGC purification method by the value of the entire retinal cell suspension (Figure 9). Regarding GFAP, the RNA ratio of RGCs purified by DMS/IMS to the whole retinal cells was 0.022; whereas the ratio of RGCs purified by TSI to whole retinal cells was 0.112. Thus, the cells purified by TSI contained more mRNA for GFAP compared to the other two methods (p=0.021). For syntaxin 1, the RNA ratio of RGCs purified by DMS, TSI, and IMS to whole retinal cells was 0.746, 0.542, and 0.447, respectively. Among the three isolation methods, the cells harvested by IMS contained the least mRNA amount for syntaxin 1 (p=0.022).

## Discussion

RGCs are a type of neuron situated in the ganglion cell layer of the inner retina. They play a crucial role in conveying visual information from photoreceptors to the brain and are damaged in glaucomatous eyes. Researchers have extensively investigated the functions of RGCs and the mechanisms of injury thereto. The ex vivo culture of RGCs may be helpful for understanding their primary responses in certain environments. However, because RGCs seldom divide in vitro or in vivo and as most RGCs have a long axon that extends into the lateral geniculate nucleus, isolating and culturing primary RGCs are very difficult.

The TSI method developed by Barres et al. [6] is widely used for RGC purification. Although the purity of TSI-isolated RGCs is more than 90%, the presence of other types of cells should not be ignored. Because RGCs actively interact with other retinal cells, the presence of a small number of contaminated cells can greatly influence experimental results. In addition, TSI requires highly skilled hands, and maintaining a constant yield is difficult. During the second panning process, each plate is gently swirled to ensure all cells have access to the surface of the antibody-coated plate and to hold the cells with relatively high affinity to anti-Thy 1 antibody. Because the binding affinity between RGCs and anti-Thy 1 antibody is not strong, plate swinging may in part cause the variable TSI yield. In this investigation, we verified the characteristics of non-RGC cells sparsely mixed with RGCs harvested by TSI. These large star-shaped cells were highly GFAP-positive and appeared to be astrocytes. Because some glial cells and lymphocytes may express Thy 1 surface antigen [6], these types of cells may contaminate RGC samples. Although the number of contaminating cells was relatively small (less than 5%), these large glial cells with rich cytoplasm could have substantially influenced surrounding RGCs.

The DMS method introduced by Shoge et al. [11] is faster and less complicated than the TSI method. Although the DMS method has the further advantage of a more stable yield after RGC purification compared with the TSI method, the purity of DMS-isolated RGCs is much lower than that of TSI-isolated RGCs. Even when retinal tissue is carefully separated and cells are dissociated into a single cell suspension, RGCs can stick to other cells. As a result of this study, non-RGC cells mixed with RGCs purified by DMS appeared to be amacrine cells. These small cells had immunoreactivity against syntaxin 1. Because we used a column optimized for cells less than 30 μm, the large glial cells may not have been selected.

To maximize the purity of harvested RGCs, we combined the TSI and DMS methods in the IMS method. At the first immunopanning step, cells reacted to antimacrophage antibody were depleted; at the second magnetic separation step, cells bound to anti-Thy 1 antibody were finally selected. RGCs isolated by IMS exhibited purity of more than 95%. As determined with immunofluorescence and western immunoblots, IMS-harvested RGCs were rarely contaminated by astrocytes or amacrine cells. Several previous reports used a two-step magnetic purification process [18,19]. At the first magnetic separation, cells that had not reacted to antimacrophage antibody were negatively selected; at the second magnetic separation, cells that were bound to anti-Thy 1 antibody were positively selected. The basic concept of a negative selection followed by a positive selection is similar to TSI and IMS. However, the cells reacted to antimacrophage antibody produce clots that block the drain inside the column.

**Figure 10:**
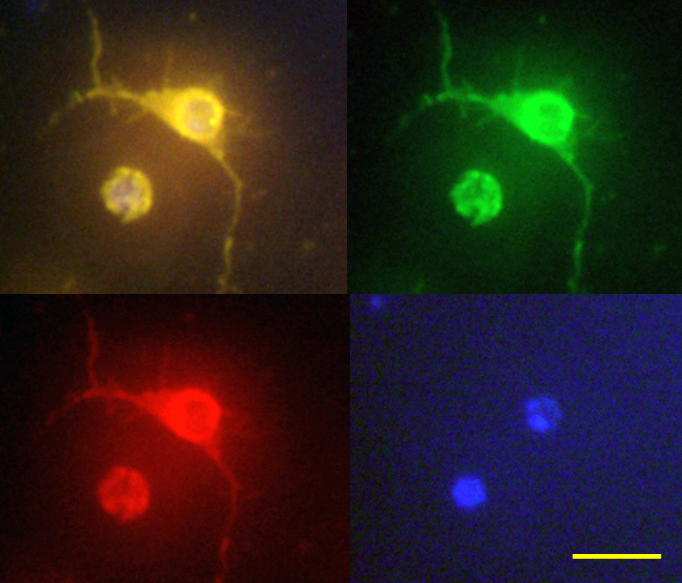
Retinal ganglion cells derived from newborn mouse retina by the immunopanning-magnetic separation method. They were labeled for thymocyte differentiation antigen 1.2 (Thy 1.2; right upper with green fluorescence) and Nestin (left lower with red fluorescence). Nuclei were counterstained with 4',6-diamidino-2-phenylindole (right lower with blue fluorescence). The left upper image is a merged image. Scale bar: 50 μm.

This is our first report outlining the isolation of primary mouse RGCs by IMS. Primary mouse RGCs have a round nucleus and abundant cytoplasm. In addition, they are characterized by stretched-out axons and multiple dendrites (Figure 10). We cultured these cells for up to eight days after isolation, and they maintained their morphological characteristics until that time. However, when the RGCs were obtained at postnatal 5 days, the cells did not maintain their RGC-like morphology after four days of culture (data not shown). Thus, we decided to purify the RGCs at postnatal 1 to 4 days.

Among mice with an Institute of Cancer Research (ICR) background, in this investigation, we used Crl:CD-1 mice (originating from Charles River Laboratories International, Wilmington, MA), which have been widely used in numerous animal studies, as these mice have good reproductive performance and a fast growth rate. Though some outbred albino mice are known to develop retinal degeneration as a result of carrying the autosomal recessive mutation of *Pdebrd1*, Cr:CD-1 mice do not show any retinal changes over 13 weeks [20]. In addition, even in some albino mice strains, which present retinal degeneration, photoreceptor cells are selectively affected rather than RGCs. Thus, the strain of mice was presumed not to influence the results of our study.

In summary, this study demonstrates that primary RGCs can be purified from newborn mice by the IMS method, combining the benefits of the TSI and DMS methods. This isolation method may provide a good experimental system for studying glaucoma in vitro.
